# Self-care or assisted PD: development of a new approach to evaluate manual peritoneal dialysis practice ability

**DOI:** 10.1080/0886022X.2022.2108448

**Published:** 2022-08-05

**Authors:** Jiaying Huang, Aiping Gu, Na Li, Yanna He, Weizhen Xie, Wei Fang, Jiangzi Yuan, Na Jiang

**Affiliations:** Department of Nephrology, Renji Hospital, School of Medicine, Shanghai Center for Peritoneal Dialysis Research, Shanghai Jiaotong University, Shanghai, PR China

**Keywords:** Peritoneal dialysis, PD practice ability, self-care PD, assisted PD, barriers to self-care PD

## Abstract

**Background:**

Peritoneal dialysis (PD) is a home-based therapy which requires the patients or their caregivers to perform the practice. We aimed to develop a practical approach to evaluate PD practice ability of the patients and to identify berries to self-care PD.

**Methods:**

A structural form was designed comprising measures of physical, cognitive, and operational abilities which were required to perform manual PD independently. The evaluation was jointly conducted by a PD nurse, a nephrologist and a close family member of the patient. Patients who met all the requirements were deemed as capable of performing PD independently (self-care PD) and others were deemed as needing an assistant (assisted PD).

**Results:**

The evaluation form was applied in 280 prevalent PD patients and 33.9% of them were assessed as needing assisted PD, mainly due to physical (62.1%) or operational (66.3%) disabilities. The evaluation result was consistent with current dialysis status in 79.3% patients and it matched better in patients who performed PD with the help of an assistant (93.0 *vs.* 76.8%, *p* = 0.014). Patients who were evaluated as having barriers to self-care PD but still performed PD without an assistant were older and demonstrated higher prevalence of diabetic nephropathy and PD-related infection, lower education level, and lower serum albumin (*p* < 0.05).

**Conclusions:**

The PD practice ability assessment form is useful to identify patients with barriers to self-care PD. It provides objective information to the patients and their family to choose feasible PD practice modality, self-care, or assisted PD.

## Introduction

Peritoneal dialysis (PD) is a successful kidney replacement therapy for patients with kidney failure. It presents comparable patient survival rate with hemodialysis (HD). Moreover, PD shows a number of social and therapeutic advantages over HD, including fewer hospital visits, improved quality of life, avoidance of hemodynamic disturbance, prolongation of residual kidney function, etc. [1] PD was introduced to China more than 60 years ago and the number of PD patients in China has grown to be the first in the world now [2].

As a home-based therapy, PD relies on the patient’s own efforts to complete the dialysis tasks. Touch contamination is a common cause of PD-related infection, which is mainly responsible for PD technique failure as well as poor patient outcome [3,4]. So, it is extremely important for the patients to perform dialysate exchanges correctly and aseptically. However, there is no established tool for PD practice ability evaluation. In most studies, barriers to conduct PD independently, including physical and cognitive impairments, were defined based on the subjective opinions of the clinicians or nurses [5,6]. Thus the evaluation results of PD practice ability of the same patient may vary if it was conducted by different evaluators.

In this study, we designed a structural and practical evaluation form to assess manual PD practice ability for the patients. It was used to evaluate the feasibility of self-care PD and decide whether an assistant is needed (assisted PD). We applied the form in prevalent PD patients and compared the evaluation results with their current dialysis status.

## Methods

The study protocol was reviewed and approved by the ethic committee of Renji Hospital, Shanghai Jiaotong University School of Medicine (approving letter No.: KY2021-147-B).

### PD practice ability assessment form

The PD practice ability assessment form was designed and established by a group of experienced nephrologists and PD nurses. Two PD nurses with working experience of longer than 10 years were responsible to draft the form separately. The evaluation items were designed based on the specific requirements to perform PD manually following standard operation procedures, including items preparation, solution exchanges, self-hygiene, communication with others, etc. Then, the two versions of assessment form were discussed point by point by the two nurses together with three nephrologists who were specialized on PD to decide the final components. Each assessment point was expressed as an easy question to let the patients and their family members to fully understand the content. The assessment form was then reviewed by three PD patients (vintage >5 years) who were performing the practice manually and independently, as well as two patients’ family members who were helping with PD. It was modified further based on the feedback of the patients and caregivers.

The finalized PD practice ability assessment form comprised three separate domains to evaluate physical function, cognitive function, and operational abilities of patients. Detail evaluation items are listed in Table 1.

**Table 1.** PD practice ability assessment form.

Patient name:	Age:	Gender:	Date:
Evaluators:	Nephrologist:	PD nurse:	Family member:
Evaluation result:	☐ self-care PD (meet all the requirements) ☐ assisted PD (do not meet all the requirements)		
Evaluation items:	Corresponding ability for conducting PD:		Result:
*Physical function*			
Able to walk for 50 m and 10 min	Ability to walk in house to prepare items for performing solution exchange		☐ Yes ☐ No
Able to lift a bag weighted 2–3 kg	Ability to lift, hang and discard a bag of PD fluid		☐ Yes ☐ No
Able to sit for half an hour	Ability to sit for conducting a cycle of solution exchanges manually		☐ Yes ☐ No
Able to see fingers clearly without blurred vision and double images	Ability to connect and disconnect the tubes of two-bag PD solution		☐ Yes ☐ No
No severe hearing impairment	Ability to communicate with doctors and nurses		☐ Yes ☐ No
*Cognitive function*			
Able to read, count and record	Ability to record urine volume and PD ultrafiltration volume. Ability to order PD fluid		☐ Yes ☐ No
Able to use the phone	Ability to ask for help in emergency		☐ Yes ☐ No
No severe cognitive impairment, able to communicate with others	Ability to communicate with doctors and nurses		☐ Yes ☐ No
Able to express body symptoms	Ability to report body symptoms to the doctors and nurses		☐ Yes ☐ No
No severe amnesia	Ability to follow the instructions step by step		☐ Yes ☐ No
*Operational ability*			
Able to tidy up a desk	Ability to tidy up a working bench for performing solution exchanges		☐ Yes ☐ No
Able to do simple housework such as hanging clothes	Ability to open and clamp the tubes using clips to conduct solution exchange		☐ Yes ☐ No
No tremor and able to complete a task such as capping a pen using both hands	Ability to connect and disconnect the tubes of two-bag PD solution		☐ Yes ☐ No
Able to wash hands, wear a mask and a hat to cap the hair	Self-hygiene		☐ Yes ☐ No
Able to do small wound cleansing and dressing after education	Ability to clean and dress the exit-site		☐ Yes ☐ No

### Patients recruitment

Prevalent patients who have been maintained on PD (continuous or daytime ambulatory PD) for longer than 3 months at Renji Hospital, Shanghai Jiaotong University School of Medicine were eligible to be included to the study. Patients using cyclers were not recruited because the practice requirements for operating a PD machine were different from manual performance. Subjects who did not have completed medical record and laboratory results in the recent six months were excluded. All patients were recruited after written informed consent.

### PD practice ability evaluation

Patients were evaluated individually using the PD practice ability assessment form. Evaluation was conducted jointly by the same group of nephrologists and PD nurses, together with a close family member of the patient, including spouse, parent, or adult child. Patients who met all the requirements were deemed as competent of self-care PD. Patients who did not meet one or more of the requirements were deemed as needing assisted PD. Demographic and clinical characteristics, as well as current dialysis status (performed by patients or by assistants) were recorded.

### Statistical analysis

Data were presented as mean ± standard deviation for normally distributed variables, median (25th and 75th percentiles) for non-normally distributed variables, or number (percentage) for categorical parameters. Student *t* test was applied to compare the difference of normally distributed variables between groups. Kruskal test was conducted to compare the difference of non-normally distributed variables between groups. Chi-square test was used to compare the differences of categorical variables or percentages between groups.

All the statistical analysis was performed using SPSS software version 25 (SPSS Inc., Chicago, IL). *p* Value <0.05 was considered as statistically significant.

## Results

A total of 280 prevalent PD patients (173 males, mean age 54.7 ± 14.7 years) were recruited in this study. Demographic and clinical characteristics are shown in detail in Table 2. Briefly, median PD duration was 33.5 (13.6, 63.2) months and chronic glomerulonephritis was the leading underlining kidney disease (37.9%). Most patients (62.1%) received education up to middle school level and 22.1% of the patients experienced one or more episodes of PD-related infection.

**Table 2.** Clinical characteristics of the studied patients and divided by current dialysis-dependent status.

		Current PD-dependent status		
	Overall (*n* = 280)	Performed by patients (*n* = 237)	Performed by assistants (*n* = 43)	*p* Value
Male: Female	173:107	149:88	24:19	0.381
Age (years)	54.7 ± 14.7	51.7 ± 13.6	70.0 ± 10.8	<0.001
BMI (kg/m^2^)	24.6 ± 3.8	24.5 ± 4.0	25.3 ± 3.4	0.254
Primary kidney diseases (*n* [%])				0.079
CGN	106 (37.9%)	96 (40.5%)	10 (23.3%)	
Diabetic nephropathy	47 (16.8%)	35 (14.8%)	12 (27.9%)	
Others	26 (9.3%)	22 (9.3%)	4 (9.3%)	
Unknown	101 (36.1%)	84 (35.4%)	17 (39.5%)	
Education experience (*n* [%])				
Illiterate	12 (4.3%)	8 (3.4%)	4 (9.3%)	0.013
Primary to high school	174 (62.1%)	142 (59.9%)	32 (74.4%)	
College or higher	94 (33.6%)	87 (36.7%)	7 (16.3%)	
PD duration (month)	33.5 (13.6, 63.2)	32.3 (14.0, 62.6)	36.1 (8.0, 73.2)	0.887
PD dose (l/d)	7.6 ± 2.0	7.5 ± 2.0	7.7 ± 1.8	0.621
Kt/V	2.1 ± 0.5	2.1 ± 0.6	2.0 ± 0.5	0.265
eGFR (ml/min/1.73 m^2^)	0.92 (0, 3.64)	0.92 (0, 3.72)	0.91 (0, 3.81)	0.162
Albumin (g/l)	37.7 ± 4.2	38.1 ± 4.0	35.2 ± 3.9	<0.001
CRP (mg/l)	1.38 (0.5, 4.1)	1.09 (0.5, 3.84)	5.73 (0.5, 7.7)	0.057
Phosphorus (mmol/l)	1.7 ± 0.5	1.7 ± 0.5	1.5 ± 0.4	0.006
PD-related infection (*n* [%])	62 (22.1%)	50 (21.1%)	12 (27.9%)	0.322

Estimated glomerular filtration rate (eGFR) was calculated as an average of the creatinine and urea clearances by 24-h urine.

CGN: chronic glomerulonephritis; PD: peritoneal dialysis.

Data were presented as mean ± SD for normally distributed variables, median (25th and 75th percentiles) for non-normally distributed variables, or number (percentage) for categorical parameters. Differences between two groups were assessed by the independent samples t-test for normally distributed variables, or Kruskal test for non-normally distributed variables, or chi-square test for categorical variables.

A total of 185 patients (66.1%) were deemed as capable of self-care PD based on the PD practice ability assessment form, whilst 95 patients (33.9%) were assessed as needing assisted PD. The evaluation results were consistent with current dialysis status, performed by patients or by assistants, in 222 (79.3%) patients (Table 3).

**Table 3.** PD practice ability evaluation results in relation to current dialysis-dependent status.

		Current PD-dependent status		
	Overall (*n* = 280)	Performed by patients (*n* = 237, 84.6%)	Performed by assistants (*n* = 43, 15.4%)	*p* Value
Evaluation results	Self-care PD (*n* = 185, 66.1%)	182	3	–
	Assisted PD (*n* = 95, 33.9%)	55	40	
Matching percentage	79.3%	76.8%	93.0%	0.014

PD: peritoneal dialysis.

Chi-square test was conducted to compare matching percentages between the two groups.

As shown in Table 4, for patients who were evaluated as needing assisted PD, operational disabilities were the most common barriers presented in 66.3% patients. Of 49.5% patients were unable to care for the exit-site (small wound cleansing and dressing) and 38.9% of them were unable to perform solution exchanges (conduct housework such as hanging clothes). Physical function impairments were seen in 62.1% patients, with reduced arm strength (unable to lift a 2–3 kg bag) and poor vision (unable to see fingers clearly) as the most common symptoms presented in 30.5% patients. Moreover, 34.7% patients showed cognitive barriers with severe amnesia as the leading impairment (17.9%). Detail evaluation results are demonstrated in Table 4.

**Table 4.** Physical, cognitive, and operational barriers in patients deemed as needing assisted PD and divided by their current dialysis-dependent status.

		Current PD-dependent status		
Evaluation items	Overall (*n* = 95) (%)	Performed by patients (*n* = 55) (%)	Performed by assistants (*n* = 40) (%)	*p* Value
*Physical function*	59 (62.1)	28 (50.9)	31 (77.5)	0.008
Able to walk for 50 m and 10 min	20 (21.2)	3 (5.5)	17 (42.5)	<0.001
Able to lift a bag weighted 2–3 kg	29 (30.5)	5 (9.1)	24 (60.0)	<0.001
Able to sit for half an hour	10 (10.5)	4 (7.3%)	6 (15.0)	0.226
Able to see fingers clearly without blurred vision and double images	29 (30.5)	18 (32.7)	11 (27.5)	0.585
No severe hearing impairment	11 (11.6)	4 (7.3)	7 (17.5)	0.193
*Cognitive function*	33 (34.7)	15 (27.3)	18 (45.0)	0.073
Able to read, count, and record	13 (13.7)	4 (7.3)	9 (22.5)	0.033
Able to use the phone	9 (9.5)	2 (3.6)	7 (17.5)	0.033
No severe cognitive impairment, able to communicate with others	3 (3.2)	0 (0)	3 (7.5)	0.071
Able to express body symptoms	4 (4.2)	4 (7.3)	0 (0)	0.136
No severe amnesia	17 (17.9)	7 (12.7)	10 (25.0)	0.123
*Operational ability*	63 (66.3)	30 (54.5)	33 (82.5)	0.004
Able to tidy up a desk	16 (16.8)	1 (1.8)	15 (37.5)	<0.001
Able to do simple housework such as hanging clothes	37 (38.9)	14 (25.5)	23 (57.5)	0.002
No tremor and able to complete a task such as capping a pen using both hands	18 (18.9)	6 (10.9)	12 (30.0)	0.019
Able to wash hands, wear a mask and a hat to cap the hair	7 (7.4)	2 (3.6)	5 (12.5)	0.103
Able to do simple wound cleansing and dressing after education	47 (49.5)	17 (30.9)	30 (75.0)	<0.001

Data were presented as patient number (percentage) of those who did not meet the evaluation items. Differences between groups were analyzed by chi-square test.

A total of 237 patients were performing manual PD by themselves and 43 patients were helped by assistants. Patients were consequently divided into two groups based on their current PD status (performed by patients or by assistants, Table 2). Patients who performed PD with the help of caregivers were older and demonstrated lower education level and lower serum concentration of albumin and phosphorus (*p* < 0.05, Table 2). PD practice ability evaluation results showed higher percentage of consistence with current dialysis status in patients who were helped by assistants than those performing PD by themselves (93.0 *vs.* 76.8%, *p* = 0.014, Table 3).

For patients who were deemed as needing assisted PD, physical dysfunction (77.5 *vs.* 50.9%, *p* = 0.008) and operational disabilities (82.5 *vs.* 54.5%, *p* = 0.004) were more common in patients receiving help from assistants than those who performing PD by themselves. The PD ability evaluation results of the two groups are listed in Table 4.

In patients who performed PD independently, 55 patients (23.2%) were deemed as needing an assistant. They were older and demonstrated higher prevalence of diabetic nephropathy, lower education level, and lower serum albumin concentration (*p* < 0.05, data not shown). Moreover, patients who were evaluated as having barriers to self-care PD but still performing PD by themselves showed higher prevalence of PD-related infection, including peritonitis, tunnel, and exit-site infection (32.7 *vs.* 17.6%, *p* = 0.016).

## Discussion

PD requires the patients to conduct dialysate exchanges at home. Incorrect practice will result in touch contamination, which is a leading cause of PD-related infection [4]. Thus, it is important to evaluate the patient’s practice ability before entering PD program and regularly during follow-up. To our knowledge, there was no validated tool to assess PD practice ability and evaluate whether an assistant is needed.

MATCH-D, Method to Assess Treatment Choices for Home Dialysis, is a standardized tool to assess the feasibility of conducting dialysis at home (http://homedialysis.org/match-d) [7]. However, the scale mixes the contraindications to PD and barriers to self-care PD together. Farragher et al. used a Comprehensive Geriatric Assessment (CGA) form in incident PD patients over 50-year old to measure the functional disability, frailty, and cognitive impairments. However, the findings of CGA only weakly correlated with PD dependency status [8]. Bevilacqua et al. developed a PD assist selection criteria for patients undergoing continuous cycler PD [9]. It is not applicable in developing countries like China, where the majority of PD patients are performing manual exchanges.

In this study, we designed a checklist form which comprised 15 items of measures covering physical, cognitive and operational requirements for performing manual PD independently. Functional and cognitive impairments are highly prevalent in patients with kidney failure and are independent predictors for adverse health outcomes [10]. In addition, in PD patients, physical barriers and cognitive dysfunction are shown to be associated with increased risk of PD-related peritonitis after adjusting for confounders [11,12]. Thus, routine screening for physical and cognitive impairments is recommended to be included as part of assessment for maintenance PD [13].

We demonstrated that PD ability evaluation results matched well with current dialysis status in prevalent PD patients, indicating it is useful to screen out patients with barriers to self-care PD. It is noted that some patients (*n* = 55, 23.2%) with impairments based on the evaluation form were still performing PD by themselves at home. This finding was similar to a previous report from Farragher et al. [8] It may be explained by the fact that the evaluation tool was sensitive to identify barriers but could not indicate the severity of deficits identified, which might impact the patient’s ability to complete the tasks. So, we will need to modify the form further to improve its accuracy. Moreover, we note that a patient’s choice of dialysis modality, self-care or assisted PD, was not influenced by their practice ability only [6]. Family support is a pronounced factor contributing to PD feasibility in impaired patients [5]. There are very few nursing houses providing PD service in China and so assisted PD is usually supported by family members [14]. At our center, a number of patients with barriers could not find a family member or afford to hire an assistant to help with solution exchanges and so they continued to perform PD even after they had experienced a decline in their abilities. These patients showed higher prevalence of PD-related infection than those who were robust to perform PD independently. The checklist can help the clinicians to identify this subset of patients. So, they could be retrained in PD practice skills and, more importantly, be helped to secure assistance.

On the other hand, we found three patients deemed as capable of performing PD independently were receiving PD assistance. One patient was assisted by his wife who was very willing to do the tasks for him. The other two patients were afraid to perform PD even though they were in fact competent to do it. So, utilization of the assessment form may help these patients getting self-confidence by providing a direct evaluation result of their practice ability.

Operational disability and physical impairment were the main gaps for self-care PD based on the assessment results in this study. The results were consistent with the findings from the study of Oliver et al. [5] Given the chronic disease status, comorbidities, relative old age of the PD patients, most of these barriers cannot be overcome by repeated training or medicine therapy. Assisted PD provided by nurses, family members or caregivers is reported to be an effective dialysis modality for these patients [15,16]. Compared to self-care PD, assisted PD even shows advantages of reducing PD-related infection and increasing PD utilization [17]. So, assisted PD is now advocated in a number of countries and areas [18,19]. Recently, a group of European nephrologists are calling for increased and equal access of assisted PD. They suggest taking actions including education of kidney healthcare teams about the advantages of PD, education of and discussion with patients and their families as they approach the need for dialysis, and engagement with policy makers and healthcare providers to develop and support assistance for PD [19].

A strength of the assessment form established in our study is that it is able to identify the specific barrier which does not allow for self-care PD. Decreased manual dexterity (exit-site cleansing and solution exchange), reduced arm strength, and poor vision were demonstrated as leading weaknesses in our patients. In consideration of limited resources, nephrologists could design personalized assisted PD regimen for each patient to break through these gaps after detail evaluation.

Patients who were receiving PD assistance or deemed as unable to perform PD independently were older and had a lower education level and lower serum albumin concentration. It is reasonable that elderly patients normally present with multiple physical, cognitive, and operational dysfunctions [17,20]. Lower education level is reported to be an independent risk factor for cognitive impairment in PD patients, which may directly reduce self-care PD feasibility [21]. Serum albumin is a biomarker of nutrition and also a known determinate of dialysis patient survival [22]. So, low serum albumin concentration indicated poor nutritional and functional status of patients who were not competent to perform PD.

PD duration is another factor which may impact the patient’s ability to perform PD. In this study, we failed to find any difference in PD duration between patients evaluated as capable of self-care PD or needing assistance. There was either no difference in PD vintage between patients who were performing PD by themselves and those already receiving assisted PD. It may be related to the fact that the study was a cross-sectional one conducted in prevalent PD patients. So, we cannot observe the changes of practice abilities of these patients along with time on PD. In a review of the patients’ previous dialysis modalities, we found that 25 patients who were initially able to perform PD independently lost their ability after years of time on PD (PD duration of 37.2 [8.0, 86.4] months). Another interesting finding was that 19 patients who were receiving assisted-PD at the start of PD demonstrated competent ability for self-care PD when participating in this study (PD duration of 21.4 [5.9, 42.8]). One reason for this phenomenon is that repeated training and practice can improve the abilities required for performing PD. Another possible explanation is that the patients’ physical function increased after treated with PD for a period of time.

There are a few limitations of this study that need to be mentioned. First of all, this was a cross-sectional study with a relatively small sample size. The PD ability assessment form was applied in prevalent patients only. So, the validity of the form needs to be evaluated in a prospective trial. Its functions in choosing best modality at the initiation of PD, as well as in identifying the need for changing modality during follow-up, remain to be determined. Second, the evaluation form is designed as a question-based checklist without a directly assessment of practice skills, which may provide more objective information of a patient’s practice ability. Another limitation of the study is that we did not evaluate the association of socioeconomic status with the patient’s ability to perform PD. Since we found that patients who were unable to perform PD independently demonstrated lower education level and serum albumin concentration, socioeconomic status may be a potential confounder here.

In conclusion, we designed a PD practice ability assessment form to evaluate the physical, cognitive and operational functions which were required for performing manual PD independently. It can be used in PD patients to identify barriers to self-care PD and decide whether an assistant is needed. Utilization of this assessment form in clinical practice may contribute to lowering PD-related infection caused by incorrect exchange practice.

## Data Availability

The datasets used and analyzed during this study are available from the corresponding author on reasonable request.
